# Spatial and temporal population dynamics of male and female *Aedes albopictus* at a local scale in Medellín, Colombia

**DOI:** 10.1186/s13071-021-04806-2

**Published:** 2021-06-08

**Authors:** Carolina Camargo, Catalina Alfonso-Parra, Sebastián Díaz, Diego F. Rincon, Luis Felipe Ramírez-Sánchez, Juliana Agudelo, Luisa M. Barrientos, Sara Villa-Arias, Frank W. Avila

**Affiliations:** 1 Max Planck Tandem Group in Mosquito Reproductive Biology, Universidad de Antioquia, Complejo RutaN, Calle 67 #52-20, Laboratory 4-166, 050010 Medellín, Antioquia Colombia; 2 Instituto Colombiano de Medicina Tropical, Universidad CES, 055450 Sabaneta, Antioquia Colombia; 3 Centro de Investigación Tibaitatá, Corporación Colombiana de Investigación Agropecuaria (AGROSAVIA), 250047 Mosquera, Cundinamarca Colombia

**Keywords:** *Aedes albopictus*, *Aedes aegypti*, Population ecology, Male–female distribution

## Abstract

**Background:**

Diseases transmitted by invasive *Aedes aegypti* and *Aedes albopictus* mosquitoes are public health issues in the tropics and subtropics. Understanding the ecology of mosquito vectors is essential for the development of effective disease mitigation programs and will allow for accurate predictions of vector occurrence and abundance. Studies that examine mosquito population dynamics are typically focused on female presence or total adult captures without discriminating the temporal and spatial distribution of both sexes.

**Methods:**

We collected immature and adult mosquitoes bimonthly for 2 years (2018–2019) in the Medellín Botanical Garden. Collection sites differed in proximity to buildings and nearby vegetation, and were classified by their overhead vegetation cover. We used linear mixed models (LMMs) and Spatial Analysis by Distance Indices (SADIE) to assess the spatial distribution of *Ae. aegypti* and *Ae. albopictus*. Using our *Ae. albopictus* captures exclusively, we assessed (1) the spatial and temporal distribution of males and females using SADIE and a generalized linear mixed model (GLMM), (2) the relationship between climatic variables/vegetation coverage and adult captures using GLMMs and LMMs, and (3) the correlation of male and female size in relation to climatic variables and vegetation coverage using LMMs.

**Results:**

Spatial analysis showed that *Ae. aegypti* and *Ae. albopictus* were distributed at different locations within the surveilled area. However, *Ae. albopictus* was the predominant species in the park during the study period. Adult *Ae. albopictus* captures were positively correlated with precipitation and relative humidity, and inversely correlated with temperature and wind speed. Moreover, we observed a spatial misalignment of *Ae. albopictus* males and females—the majority of males were located in the high vegetation coverage sites, while females were more evenly distributed. We observed significant associations of the size of our adult *Ae. albopictus* captures with precipitation, temperature, and wind speed for both sexes and found that overhead vegetation cover influenced male size, but observed no effect on female size.

**Conclusions:**

Our work elucidates the differential dynamics of *Ae. albopictus* males and females, which is pivotal to develop accurate surveillance and the successful establishment of vector control programs based on the disruption of insect reproduction.

**Graphic Abstract:**

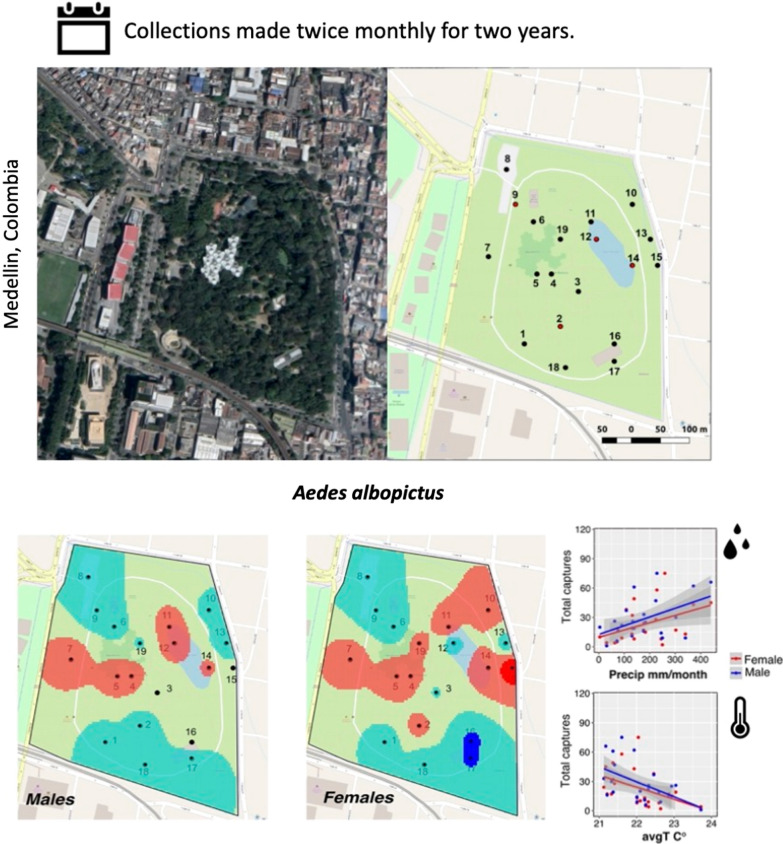

**Supplementary Information:**

The online version contains supplementary material available at 10.1186/s13071-021-04806-2.

## Background

*Aedes aegypti* and *Ae. albopictus* mosquitoes are invasive species responsible for the dissemination of viruses that adversely affect human health, including the dengue [1], Zika [2], and chikungunya viruses [3]. Both species are present throughout the tropics and sub-tropics, and their populations often co-occur in the same habitats [4]. *Aedes albopictus* is further distributed in more temperate regions due to the ability of this species to tolerate low temperatures, diapause during winter months, and lay eggs that are resistant to desiccation [5–9]. Both *Ae. aegypti* and *Ae. albopictus* have successfully colonized urban- and peri-urban environments and are anthropophilic [10–13]. As territory habitable by *Ae. aegypti* and *Ae. albopictus* continues to expand [14, 15], the incidence of diseases disseminated by these vectors is expected to increase, as has been previously observed [16, 17].

In Colombia, diseases spread by *Ae. aegypti* and *Ae. albopictus* are continuing public health concerns [18, 19]. For example, more than 23 million Colombians—nearly half the country’s population—live in areas considered at risk for dengue infection [19], and dengue epidemics occur every 3–4 years [19–21]. In Medellín, Colombia’s second largest city, *Ae. aegypti* was the predominant species until 2011, when the presence of *Ae. albopictus* was first detected [22]. Since that time, *Ae. albopictus* populations have been established in several areas of Medellín (Secretaria de Salud Medellín, unpublished data). Overlapping ecological niches of these two species can result in the competitive displacement of *Ae. aegypti* by *Ae. albopictus* or the stabilization and co-existence of both species [23, 24].

*Aedes aegypti* and *Ae. albopictus* differ in their host feeding preferences [25] as well as their disease transmission abilities [3], factors that can influence disease transmission rates. Additionally, interactions between *Ae. albopictus* and *Ae. aegypti* populations have implications for mosquito control programs that introduce transgenic [26] or *Wolbachia*-infected [27] *Ae. aegypti* into the field. Beginning in 2017 in Medellín, the World Mosquito Program began releasing *Wolbachia*-infected *Ae. aegypti* males and females to replace native populations [28], as artificial infection of *Ae. aegypti* females by *Wolbachia* blocks transmission of some viruses [29, 30]. The continued invasion of Medellín by *Ae. albopictus* is likely to influence the successful implementation of this program. Understanding the population dynamics of *Ae. albopictus* where control programs are implemented may identify factors that influence the establishment of *Wolbachia*-infected *Ae. aegypti* in areas where *Ae. albopictus* populations exist. Further, elucidation of the spatial dynamics of *Aedes* males and females will aid control programs that exclusively release males [26, 31]. To date, most emphasis has been placed on examining *Ae. aegypti* population dynamics, but little information regarding *Ae. albopictus* has been reported.

Parks and other green recreational areas in urban settings have been shown to sustain populations of medically relevant mosquitoes [13, 32–34]. Medellín is a municipality that carries a heavy burden for tropical diseases disseminated by *Aedes* mosquitoes [18, 35], but studies that assess mosquito populations in parks and other high vegetation areas of the city are limited. Although environmental factors influence mosquito population density at both the adult and larval level in tropical urban parks [32–34], each study area carries a unique combination of factors that can influence local population structures. Further, factors that influence male and female mosquito distribution within a specified area are largely unknown. In the present study, we surveyed adult and immature mosquitoes twice monthly over a 2-year period in the Medellín Botanical Garden, a centrally located park with abundant vegetation in an area of the city with *Ae. aegypti* and *Ae. albopictus* populations (Secretaria de Salud Medellín, unpublished data). Our aim was to evaluate the spatial and temporal population dynamics of these species, assess how climatic variables and vegetation coverage influence adult captures, and determine whether *Aedes* males and females differed in their local distributions around the park. As we found that *Ae. albopictus* was the predominant species during the entirety of the study, accounting for ~ 95% of adult captures, we used our *Ae. albopictus* data exclusively to assess the influence of environmental variables on adult captures, size, and male–female distribution within the study area.

## Methods

### Study area

**Fig. 1:**
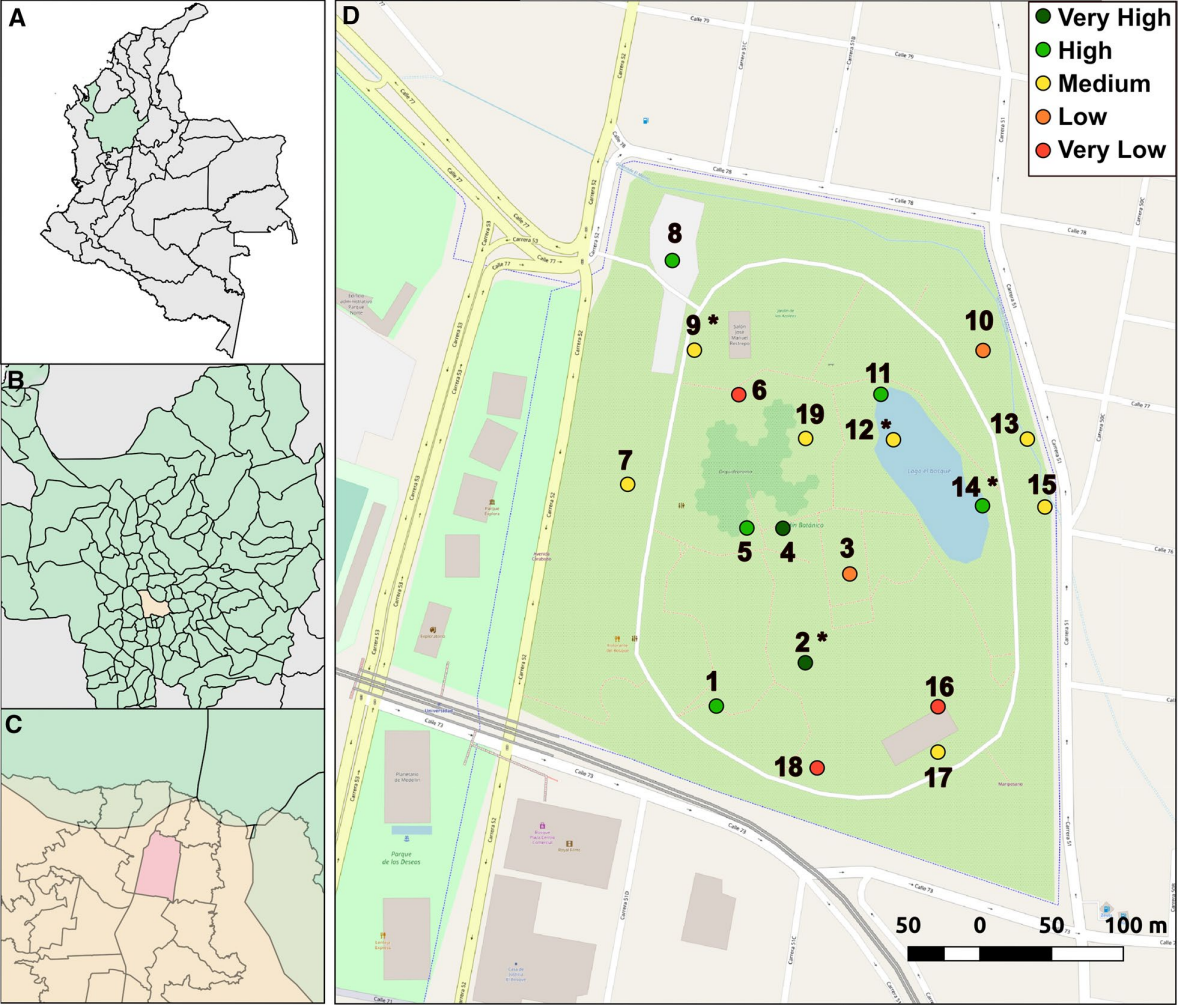
Location of the Medellín Botanical Garden. Map of Colombia with the department of Antioquia in green (**A**), Antioquia with the city Medellín in yellow (**B**), Medellin with the location of the botanical garden in pink (**C**). Larvae and adult collection sites are shown in (**D**). Adults were collected at all sites shown, and larvae at sites marked with an asterisk (*). Colors for each site represent the level of vegetation coverage (upper right corner)

This study was conducted in the Jardín Botánico de Medellín (Medellín Botanical Garden; Fig. 1A–C), which has an area of 14 ha and is located near the city center (6° 16′ 25″ N and 75° 33′ 47″ W). At 1400 m above sea level, the Medellín Botanical Garden has a subtropical semi-humid climate with vegetation consisting of tropical flora including palms, bromeliads, orchids, ferns, and cycads [36]. The garden is home to various species of birds, small mammals (cats, monkeys, and squirrels), and reptiles [36]. The area immediately around the garden consists of residential housing, businesses, and other public spaces (a park, a museum, and a university), and is adjacent to the city metro line. The Medellín Botanical Garden receives an estimated 130,000 people each month [36].

### Mosquito collection

Larvae and adult mosquitoes were collected from January 2018 to December 2019. Sampling was conducted every 2 weeks in the late morning (9 am–12 pm). Adult collections were made at 19 locations within the park (Fig. 1D), employing both human landing catch and sweep nets to capture adults. The selection of the sites was intended to represent different ecological settings, ranging from sites near buildings, squares with few plants or trees, and sites with a high density of bushes, plants and trees (collection sites are described in Additional file 1: Table S1). To ensure that collections were made at the same locations, the geographic coordinates were recorded. The average distance between sampling points was 171.22 m, with a minimum distance of 29 m and a maximum distance of 381 m (Additional file 1: Table S1). Four researchers remained at each sampling site for 5 min. Researchers typically wore short-sleeved shirts and long pants. Captured adults were aspirated into 50 ml conical tubes and labeled with the collection site identification code. Larvae were collected at four locations within the park (Fig. 1D; Additional file 1: Table S1). These four sites represented accessible areas that had natural containers (e.g., tree holes) that consistently accumulated water; when present, 20–100 ml of water was collected, depending on the volume available. Adult mosquitoes were brought to the laboratory to identify sex and species using published keys [37, 38]. Wing lengths of adult captures were measured as in [39] to estimate body size; the right wing of each adult was measured from the apical notch to the axillary margin (excluding the fringe). Larvae from each collection site were transferred to 500 ml containers with 100 mL dH_2_O and given a pinch of fish food (TetraMin) until pupation. Pupae were transferred to 5 ml tubes and the species and sex of each specimen was determined upon eclosion.

### Spatial analysis of *Aedes* mosquitoes

We first analyzed the two main response variables of our models—adult captures and larvae collections—to determine the probability distribution that fit the two datasets including normal, negative binomial, and Poisson distributions. The Akaike information criterion (AIC) was used to determine the best distribution that fit the data; the lowest AIC value corresponds to the best fitted distribution. We found that for our adult and larval collections, the lowest AIC corresponded to a normal distribution (adult AIC: Poisson 908.46, negative binomial 255.19, normal 241.23; larvae AIC: Poisson 3570.21, negative binomial 322.46, normal 305.09). Therefore, we used two separate linear mixed models (LMM; one for adult and one for larvae) to evaluate the interaction between year and site to determine whether collections at each site were similar in both 2018 and 2019 in order to consolidate the 2-year dataset for the spatial distribution analysis. To account for the variation in the number of adults and larvae collected at each site for each year, month was used as a random factor in these models. Moreover, to evaluate the proportion of male and female *Ae. albopictus* adults captured at each site in the 2 years of study, we used a generalized linear mixed model (GLMM) with a binomial distribution using the proportion of males and females as the response variable, and site and year as fixed variables to determine the interaction between these two factors. Month was also used as a random factor in these models.

We were interested in detecting patches of consistently high counts relative to the surrounding locations in order to determine whether there were sites in the park where (1) adult *Ae aegypti* or *Ae. albopictus* and (2) adult *Ae. albopictus* males and females tend to aggregate, and whether the species and sex tend to coexist or compete at certain sites within the park. We used Spatial Analysis by Distance Indices (SADIE) methodology [40, 41] to detect locations where adults were consistently aggregated (patches) or absent (gaps). SADIE calculates overall aggregation through *D*, the minimum distance to achieve regularity (i.e., the most homogeneous distribution of individuals among sampling sites) for the counts in the dataset. The quotient of *D* and the mean minimum total distance to regularity of thousands of permutations of the collected data (i.e., randomly generated alternative spatial configurations of adult counts) yields an overall aggregation index denoted as *I*_*a*_, and a *p*-value for significance with a null hypothesis of having obtained the observed distribution of counts by chance (i.e., randomness, denoted as *P*_*a*_). When *I*_*a*_ > 1, counts are considered aggregated; otherwise this is an indication of regularity. SADIE provides local indices of clustering, *v*_*i*_ for patches and *v*_*j*_ for gaps, depending on whether they are above or below (*v*_*i*_ > 1 or *v*_*j*_ < −1), or well above or well below (*v*_*i*_ > 1.5 or *v*_*j*_ < −1.5) the expectation, respectively. These cluster values are then used to calculate neighborhoods of high counts (*V*_*i*_) or low counts (*V*_*j*_).

We also tested spatial associations between *Ae. albopictus* males and females and adult *Ae. albopictus* and *Ae. aegypti* using the spatial association test provided by SADIE [42], which tests the significance of association (or disassociation) between two sets of count data, and detects locations where such association is statistically significant. The spatial association between two datasets, *X*, is given by the local index *x*_*k*_*.* If there is presence of either a patch or a gap for the two datasets, it represents a positive association at the local scale. On the other hand, a negative association, or disassociation, represents a patch for one data and a gap for the other in the same location. The overall spatial association, *X*, is the correlation coefficient between clustering local indices of two datasets and ranges from −1 to 1. Significance of *X* (Ho: *X* ≠ 0) was assessed by comparing the value obtained from the data with the quantiles derived from *X*_rand_, the overall index values of 4000 permutations (i.e., randomly generated alternative spatial configurations) of the two datasets. Contour maps of local association were constructed by the inverse distance weighting (IDW) interpolation [43] across the entire sampling region. Critical values for the contour maps were derived from the quantiles obtained in the permutations, *X*_rand_, which represent the random variate of the null hypothesis (i.e., distribution of counts obtained by chance). Values of *x*_*k*_ that were > 85% of *X*_rand_ were considered significantly associated; those < 85% of *X*_rand_ were considered significantly disassociated. To correct for spatial autocorrelation, critical values were multiplied by an inflation factor derived from the method of [44], after a second-order polynomial detrend [45].

### Analysis of overhead vegetation cover

We evaluated the correlation of adult male and female *Ae. albopictus* captures with the overhead vegetation cover of each collection site (i.e., leaves, branches, and flowers). Overhead vegetation cover of the collection sites was characterized using the %Cover application (Public Interest Enterprises, Newcastle, Australia) which converts digital photographs taken in a vertical direction to a binary image and calculates the percentage of black and white pixels. Photographs of each collection site were taken 1 m above the ground. Sites were classified into five categories based solely on the calculated overhead vegetation cover: very high (≥ 90%), high (≥ 80%), medium (≥ 70%), low (≥ 60%), and very low (≥ 40%) (Additional file 1: Table S1). For the statistical analysis, we used a LMM for the total adult captures as the response variable and a GLMM model with a binomial distribution for the proportion of males and females as the response variable. In both models, the five established categories were used as fixed variables. As we had a differing number of sites of each overhead cover classification, we accounted for this variability by using site per vegetation coverage as a random factor in the model. The number of males, females and total *Ae. albopictus* captured were used as the response variables. Mean comparison was carried out with a Tukey-test.

### Temporal analysis of *Aedes* mosquitoes and correlation with environmental variables

Differences in captures were analyzed per month per year using a LMM, with site as a random factor in the model. Adult and larvae captures were correlated with precipitation, temperature, wind speed, humidity, and atmospheric pressure per month. Measurements for these variables during the study period were obtained from the environmental station located in the Botanical Garden maintained by the Sistema de Alerta Temprana de Medellín y el Valle de Aburrá (SIATA; Early alert system of Medellín and the Aburrá Valley). Data from the environmental station is taken each minute. We used monthly averages for the purpose for this study (Additional file 2: Table S2); precipitation data was the average monthly accumulation.

### Wing size analysis

Because mosquito body size is related to female longevity and reproductive output [46, 47], we examined how *Ae. albopictus* size changed during the study period and asked whether collection site or environmental variables influenced this trait. We used different LMMs to analyze the wing sizes of captured adults; wing size was used as the response variable. We first evaluated the overall differences between the sexes and species using each as a fixed variable, and captures per month per site as a random factor in the model. We next analyzed the change in wing sizes using month and year as fixed variables and captures per site as a random factor in the model. To analyze wing size distribution within the park we used site and year as fixed variables and captures per month as a random factor in the model. We also analyzed the distribution of size across the five vegetation categories (see above), using overhead vegetation cover as a fixed variable and site per vegetation category as a random factor in the model. Finally, we developed a LMM of wing size as a function of each environmental variable assessed (precipitation average, maximum and minimum temperature, wind speed, and atmospheric pressure; Additional file 2: Table S2).

## Results

### Mosquito species collected in the Medellín Botanical Garden

From January 2018–December 2019, adult and immature mosquitoes were collected every 2 weeks. At four sites that had consistent water reservoirs (Fig. 1D), we collected 7376 larvae (5591 of which survived to adulthood). *Aedes albopictus* was the predominant species collected, accounting for 80.27% of the surviving larvae, followed by *Culex* spp. (13.92%) and *Ae. aegypti* (5.81%) (Table 1). Slightly more female larvae were collected, although both sexes were found in similar proportions (Table 1). Adults were captured at 19 different sites (Fig. 1D); 1398 adults were captured in total. *Aedes albopictus* was the predominant species (94.56%) followed by *Ae. aegypti* (4.22%). *Culex* spp. adults were rarely captured (0.14%). We were unable to identify 1.07% of the adults (Table 1) due to our inability to reliably visualize morphological markers, likely to due to specimen age; an additional 2 specimens were damaged during processing. More adult males of each *Aedes* species were collected overall (Table 1). Thus, in both our larval and adult collections, *Ae. albopictus* were the predominant species observed in the park during the study period.

**Table 1:** Larvae and adult captures in the Medellín Botanical Garden during the study period (2018–2019)

Species	Sex	Adults						Larvae					
		2018		2019		Total *N*	Total %	2018		2019		Total *N*	Total %
		*N*	%	*N*	%			*N*	%	*N*	%		
*Ae. aegypti*	Female	10	1.55	17	2.25	27	1.93	69	2.03	99	2.48	168	2.28
	Male	14	2.17	18	2.39	32	2.29	69	2.03	88	2.21	157	2.13
	Total	24	3.73	35	4.64	59	4.22	138	4.07	187	4.69	325	4.41
*Ae. albopictus*	Female	280	43.48	270	35.81	550	39.34	1013	29.86	1269	31.85	2282	30.94
	Male	336	52.17	436	57.82	772	55.22	943	27.80	1263	31.70	2206	29.91
	Total	616	95.65	706	93.63	1322	94.56	1956	57.67	2532	63.55	4488	60.85
*Total Aedes*		640	99.38	741	98.28	1381	98.78	2094	61.73	2719	68.25	4813	65.25
*Culex sp.*	Female	2	0.31	0	0.00	2	0.14	122	3.60	296	7.43	418	5.67
	Male	0	0.00	0	0.00	0	0.00	104	3.07	256	6.43	360	4.88
	Total	2	0.31	0	0.00	2	0.14	226	6.66	552	13.86	778	0.55
*Unidentified*	Female	1	0.16	11	1.46	12	0.86	–	–	–	–	–	–
	Male	0	0.00	1	0.13	1	0.07	–	–	–	–	–	–
	Unidentified	1	0.16	1	0.13	2	0.14	–	–	–	–	–	–
	Total	2	0.31	13	1.72	15	1.07	1072	31.60	713	17.90	1785	24.20
*Total all species*		644	100	754	100	1398	100	3392	100.00	3984	100.00	7376	100.00

Unidentified larvae correspond to larvae that died prior to eclosion

### Spatial analysis of *Aedes aegypti* and *Aedes albopictus* in the Medellín Botanical Garden

Because collection sites were variable (i.e., differences in vegetation, canopy cover, proximity to buildings, etc.; sites are described in Additional file 1: Table S1), we examined the spatial distribution of *Ae. aegypti* and *Ae. albopictus* within the park and determined exclusion or association sites of both populations. The majority of larvae was collected at bamboo posts and tree holes: sites 9 and 14 (44.70% and 38.73% of the total larvae, respectively) in 2018, and at sites 12 and 14 (31.96% and 56.49%) in 2019. Regarding *Aedes* species, most *Ae. aegypti* larvae were collected at site 9 (69.57%) in 2018, and at sites 9 and 14 (39.04% and 38.50%, respectively) in 2019; most *Ae. albopictus* larvae were collected at sites 9 and 14 (42.94% and 38.29%) in 2018, and at sites 12 and 14 (37.66% and 57.82%) in 2019 (Additional file 7: Figure S1A, B). We found statistically significant differences between 2018 and 2019 in the yearly average of larvae collected at each site (LMM: DF = 3, *F* = 5.7, *p* = 0.041, estimate = 55.83, SE = 43.89; Additional file 7: Figure S1A, B).

**Fig. 2:**
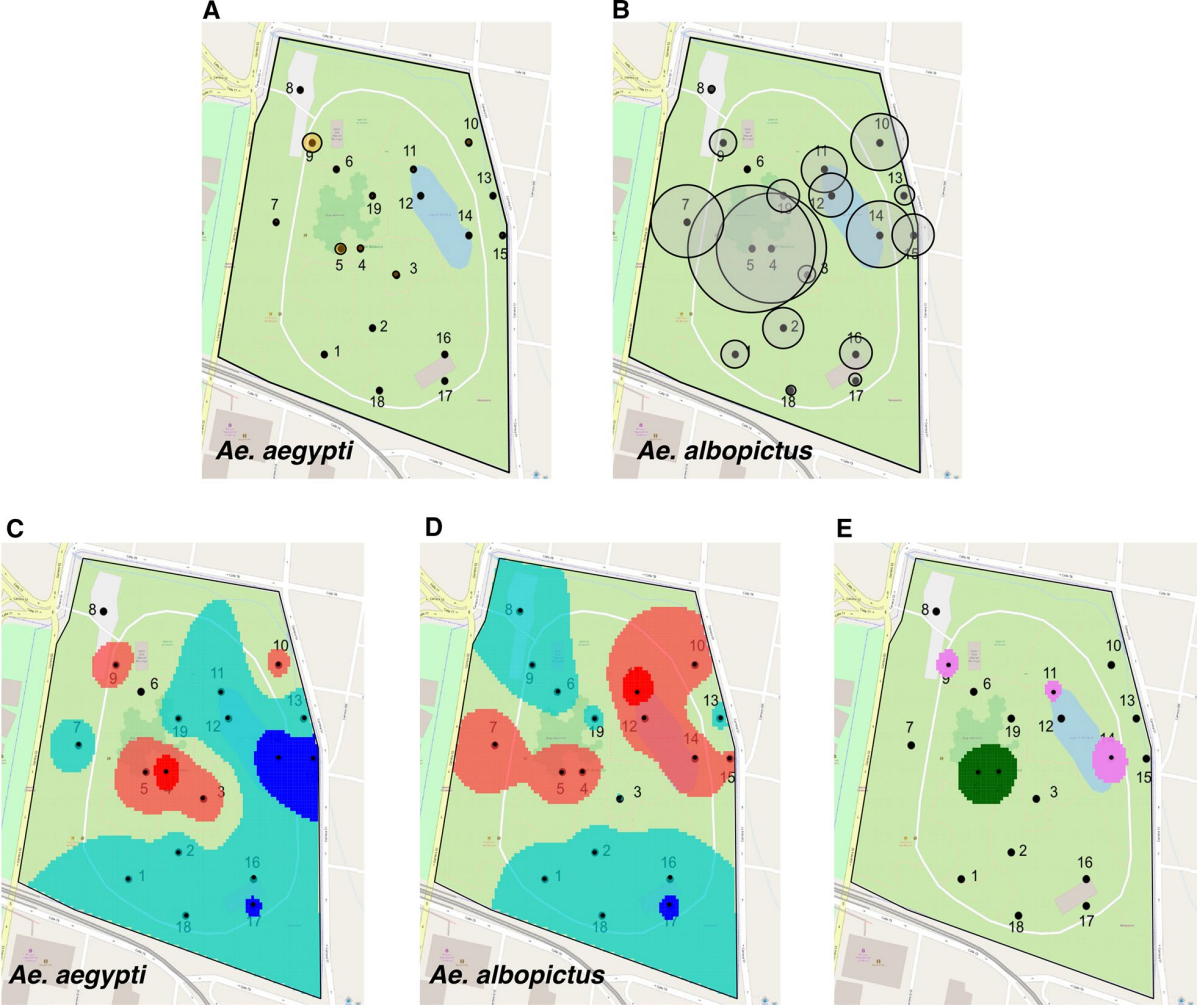
Spatial distribution of adult *Aedes aegypti* and *Aedes albopictus* in the Medellín Botanical Garden. Total captures per site for *Ae. aegypti* (**A**) and *Ae. albopictus* (**B**). Distribution pattern of *Ae. aegypti* (**C**) and *Ae. albopictus* within the park (**D**). Shaded areas represent local indices of clustering: orange above expectation (*V*_*i*_ > 1), red well above expectation (*V*_*i*_ > 1.5), green below expectation (*V*_*j*_ < −1) and blue well below expectation (*V*_*j*_ < −1.5). Map showing significant association (green) and disassociation (violet) of both species is shown in (**E**). The diameter of the bubbles in (**A**) and (**B**) is directly proportional to the counts in the respective centroid location

Although *Ae. aegypti* adults were detected in low numbers, individuals were captured at eleven sites (Additional file 7: Figure S1C, D), with most individuals captured at sites on the periphery of the park with medium and low vegetation (sites 9 and 10, respectively), and in the middle of the park with low (site 3) and high vegetation (sites 4 and 5) (Fig. 2A, Additional file 7: Figure S1C, D; Additional file 3: Table S3). *Aedes albopictus* adults were collected at all 19 sites; more than 60% were collected at sites 4, 5, 7, 10, and 14 (Fig. 2B, Additional file 7: Figure S1C, D; Additional file 3: Table S3). We observed no statistically significant differences between 2018 and 2019 in the average number of adults collected at each site (LMM: DF = 18, *F* = 1.42, *p* = 0.11, estimate = 3.25 e−02, SE = 8.44 e−02; Additional file 7: Figure S1C, D), although we observed statistically significant differences in population sizes between sites (LMM: *F* = 6.802, DF = 18, *p* < 0.001, estimate = 6.55 e + 01, SE = 1.70 e + 02; Fig. 2A, B; Additional file 7: Figure S1C, D).

We next characterized the spatial aggregation of individuals of each species, and the spatial association between *Ae. aegypti* and *Ae. albopictus* using SADIE [40, 41]. The overall index of aggregation for the mosquito populations in the park was *I*_*a*_ = 1.15 (*P*_*a*_ = 0.20) and *I*_*a*_ = 1.06 (*P*_*a*_ = 0.33) for *Ae. aegypti* and *Ae. Albopictus*, respectively, which suggests an overall moderate patchiness of both species across the sampled region that is not biologically significantly different from a random pattern. However, at a local scale, we identified individual sites where populations aggregate, forming biologically significant patches and gaps for each species (Fig. 2C, D). This departure of overall aggregation (*I*_*a*_) from local indices (*v*_*i*_) may be a result of small sample sizes or edge effects (i.e., large or small counts consistently around the sampling area)—as in our case—when local indices are more powerful at detecting nonrandom distributions [48, 49].

We observed *Ae. aegypti* aggregations at five sites and a patch with statistically significantly above-average density at site 4 (*v*_*i*_ > 1.5; Fig. 2C). There were nine areas with low densities or gaps, while sites 14, 15, and 17 had significantly below-average densities (Fig. 2C). A different local pattern was found for *Ae. albopictus*: we found biologically significant aggregations at seven sites with a statistically significantly above-average patch at site 11 (*v*_*i*_ > 1.5; Fig. 2D). There were eight sites with low density and a gap at site 17 (*v*_*j*_ = −1.5; Fig. 2D). Spatial association between *Ae. albopictus* and *Ae. aegypti* was statistically and biologically significant at sites 4 and 5 (*p* < 0.05; Fig. 2E), where both species aggregate and the majority of adults were captured (Fig. 2A, B; Additional file 7: Figure S1C, D; Additional file 3: Table S3). Statistically significant local disassociations of *Ae. aegypti* and *Ae. albopictus* were found at sites 9, 11 and 14 (*p* < 0.05; Fig. 2E); only *Ae. aegypti* was aggregated at site 9 and only *Ae. albopictus* was aggregated at sites 11 and 14. We did not observe a statistically significant disassociation (*p* = 0.5844) or association (*p* = 0.415) for the overall population across the study area but found co-existence and exclusion locally. Overall, *Ae. aegypti* and *Ae. albopictus* aggregated locally in different areas of the park and found to exclude one another in areas with high and medium vegetation at sites near the periphery of the park, although both species were also found to associate at two centrally located sites with high overhead vegetation cover.

### Spatial analysis of male and female *Aedes albopictus* in the Medellín Botanical Garden

**Fig. 3:**
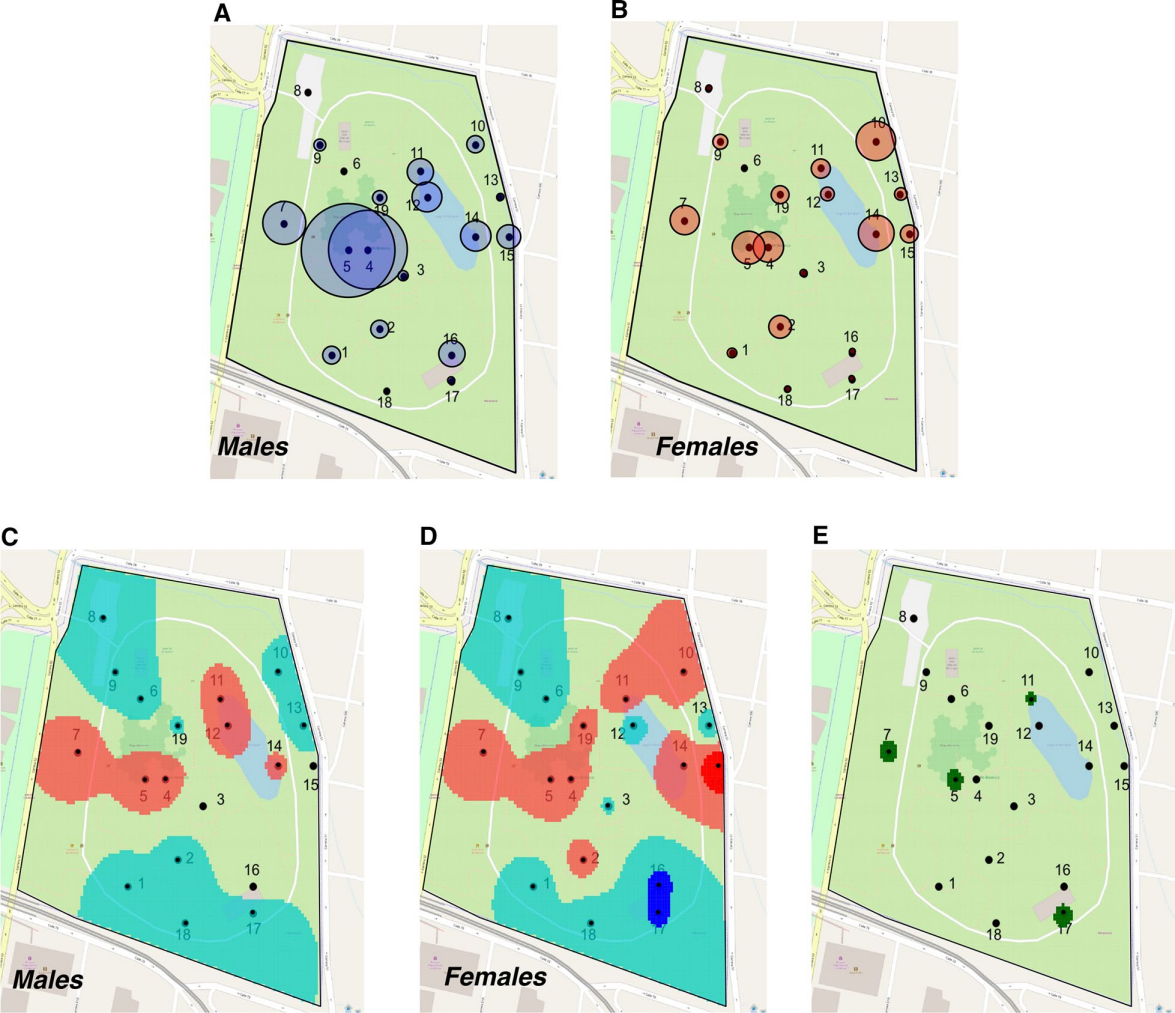
Spatial distribution of *Aedes albopictus* males and females in the Medellín Botanical Garden. Total male (**A**) and female (**B**) captures per site. Distribution pattern of *Ae. albopictus* males (**C**) and females (**D**) within the park. Shaded areas represent local indices of clustering: orange above expectation (*V*_*i*_ > 1), red well above expectation (*V*_*i*_ > 1.5), green below expectation (*V*_*j*_ < −1) and blue well below expectation (*V*_*j*_ < −1.5). Overall map showing sites with significant associations (green) of each sex is shown in (**E**). The diameter of the bubbles in (**A**) and (**B**) is directly proportional to the counts in the respective centroid location

As we collected more *Ae. albopictus* during this study (58.40% males vs. 41.60% females; Table 1), we examined *Ae. albopictus* male and female distribution within the park using our adult capture data to identify areas where they aggregate or disperse. For the combined captures at each site, the largest proportion of males were collected at sites 4 and 5 (Fig. 3A; Additional file 8: Figure S2), two sites with a high overhead vegetation cover (Fig. 1, Additional file 1: Table S1). Females had higher proportions at sites 10 and 14 (Fig. 3A, B), sites with low and high overhead vegetation cover, respectively. We observed statistically significant differences in the average male–female proportions between sites (GLMM: *F* = 13.39, DF = 18, *p* < 0.001, estimate = 4.44, SE = 12.33; Fig. 3A, B). However, the proportion of males and females collected at each site showed a similar pattern between the 2 years of study, with no statistically significant interaction between site and year (GLMM: DF = 18, *F* = 0.11, *p* = 0.73, estimate = 0.0021, SE = 0.006; Additional file 8: Figure S2).

The overall distribution of males and females was moderately patchy, but not statistically significantly different from what is expected by chance (males: *I*_*a*_ = 0.9, *P*_*a*_ = 0.63; females *I*_*a*_ = 1.18, *P*_*a*_ = 0.157). Locally, however, we found that males aggregated in fewer patches than females (Fig. 3C). Additionally, there is a statistically significant patch with a high density of females at site 15 (*v*_*i*_ > 1.5; Fig. 3D). Both sexes had low densities at seven sites. However, sites 2, 10, and 19 were occupied by females but not by males. Statistically significant gaps for females were found at sites 16 and 17 (*v*_*i*_ < −1.5; Fig. 3D). We found that both sexes were significantly associated across the sampled area (*p* = 0.016). Biologically and statistically significant local associations were observed at sites 5, 7, and 11 (Fig. 3E), where high numbers of both males and females were recorded (Fig. 3A, B). Site 17 also had a statistically significant local association, due to concomitant small counts of both sexes (Fig. 3C, D). Overall, *Ae. albopictus* males and females were observed to have different local distribution patterns in the Medellín Botanical Garden, with males aggregating at less sites than females.

We further analyzed the spatial distribution of males and females during the dry and rainy seasons of Medellín, which has two distinct periods of high and low precipitation annually (Additional file 2: Table S2). We combined data for the dry seasons (first: December–February; second: June –August) and rainy seasons (first: March–May; second: September–November). As our collections began in January 2018, analysis of the first dry season did not include data from December 2017. At a local scale, we found a similar pattern of patch and gap distribution for both males and females during the first and second dry season (Additional file 9: Figure S3A, B, E, F). However, the distribution of patches and gaps for both sexes differed between the first and second wet season. Interestingly, the pattern observed in the second wet season resembled that observed in the first dry season for both sexes (Additional file 9: Figure S3C, D, G, H). We also observed significant associations in certain areas of the park where both sexes aggregate, mainly at sites 4 and 5 (Additional file 9: Figure S3I, J, K, L) where high overhead vegetation coverage was found. Although within-year variation observed for the rainy and dry seasons makes it difficult to describe general differences, it appears that *Ae. albopictus* males and females aggregate differently in space during the year and that this distribution is influenced by weather variables.

### Overhead vegetation coverage influences *Aedes albopictus* captures

**Fig. 4:**
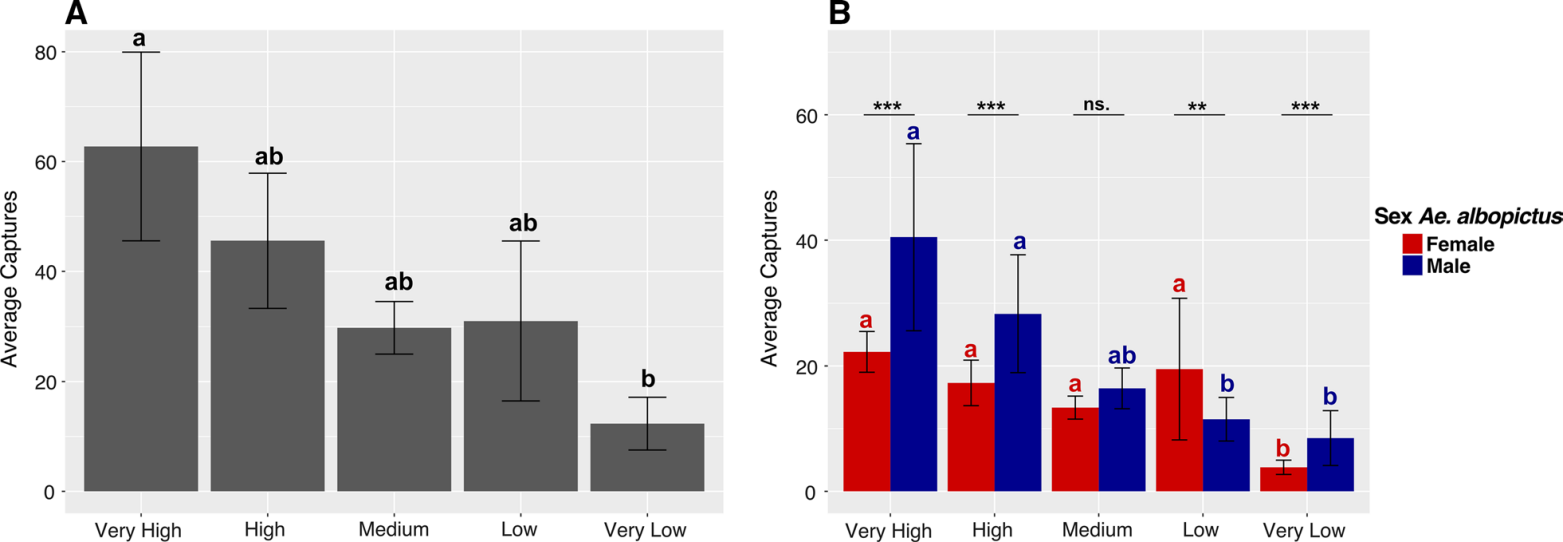
Total adults captured at sites with the corresponding vegetation coverage. Adult *Ae. albopictus* collected (average ± SE) during the 2-year study period (**A**), and total males and females collected (**B**). Different letters show significant differences between the average number of adult captures at each vegetation cover classification using a post hoc Tukey test (***p < 0.0001, **p < 0.001, *p < 0.01)

We next examined whether overhead vegetation coverage influenced *Ae. albopictus* captures, classifying each collection site by its percentage overhead vegetation cover, which ranged from very low to very high (Fig. 1D; Additional file 1: Table S1). Overhead vegetation cover had a significant effect on adult captures (LMM: *F* = 2.858, DF = 4, *p* < 0.001, estimate = 45.35, SE = 8.58; Fig. 4A). The majority of *Ae. albopictus* were captured at areas classified as sites with high or very high overhead vegetation cover (Fig. 4A). Males and females were uniquely distributed across areas that differed in vegetation cover—significantly more males were found at areas with very high or high overhead vegetation coverage sites compared to females, who were similarly distributed between sites with low to high coverage (LMM: *F* = 16.773, DF = 4, *p* < 0.001, estimate = 0.49, SE = 0.096; Fig. 4B). Thus, overhead vegetation cover appears to influence overall *Ae. albopictus* abundance in the Medellín Botanical Garden but has a greater effect on male abundance.

### Adult *Aedes albopictus* captures in relation to weather variables

**Fig. 5:**
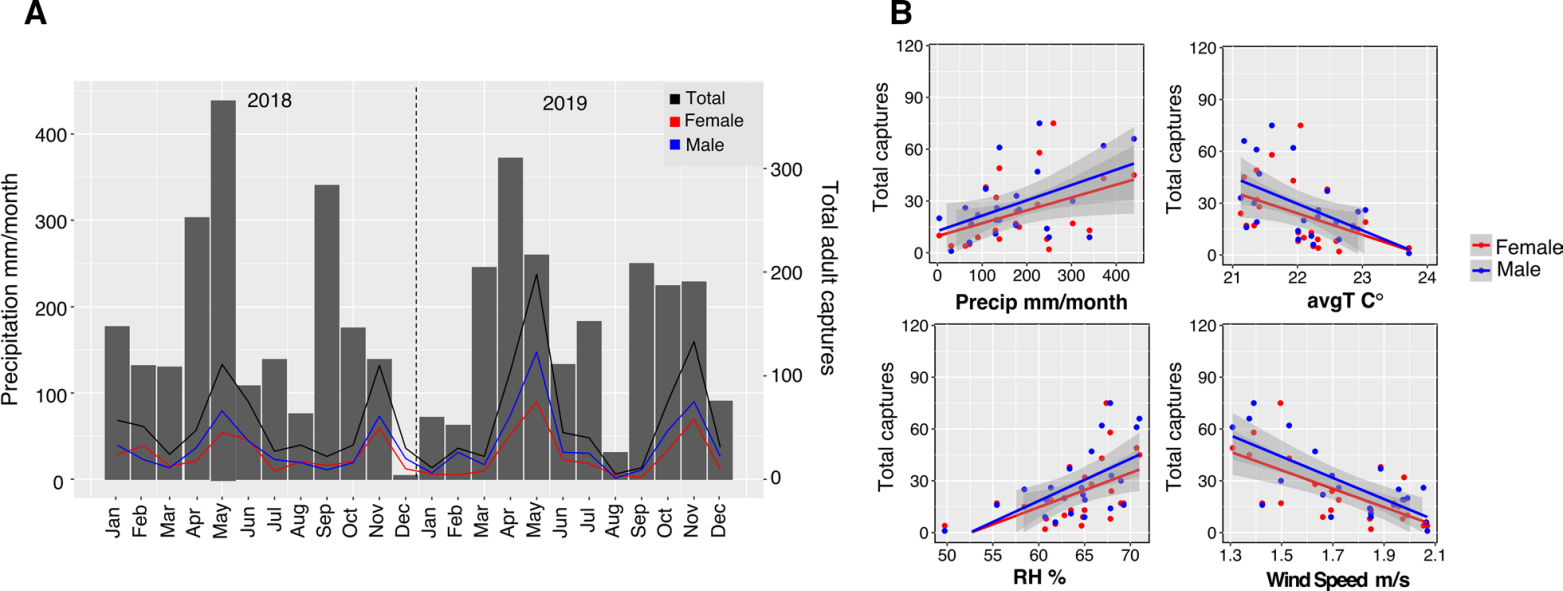
Correlation of *Aedes albopictus* adult captures with weather variables. Temporal distribution of male and female *Ae. albopictus* adult captures (**A**) in relation to precipitation during 2018 and 2019. Linear model fit between weather variables and male and female adult captures is shown in (**B**)

We examined how weather variables correlated with our monthly *Ae. albopictus* collections. Adult captures showed two distinct peaks during April–May and October–November in both 2018 and 2019, coinciding with months with the highest cumulative precipitation (Fig. 5A); we found a significant positive correlation with male and female captures during these months (LMM: *p* = 0.027; Additional file 4: Table S4). Relative humidity also had a significant positive correlation with male and female captures (LMM: *p* = 0.008; Fig. 5B; Additional file 4: Table S4). Temperature and wind speed showed a significant inverse correlation with adult captures (LMM temperature: *p* = 0.044; wind speed: *p* = 0.0002; Fig. 6B; Additional file 4: Table S4). We found no significant correlation with *Ae. albopictus* larvae collected for any evaluated environmental variable (Additional file 5: Table S5), although we also observed a significant correlation between precipitation and relative humidity with total larvae collected (LMM precipitation: *p* = 0.0045; relative humidity: *p* = 0.0145). The highest number of larvae collected occurred during months with the highest precipitation in both 2018 and 2019 (Additional file 10: Figure S4), although we also collected larvae in high numbers in January of 2019, a month with low rain levels.

### Environmental factors influence *Aedes albopictus* body size

Using wing length as a proxy for body size [39], we observed a statistically significant differences in size between species (LMM: DF = 1, *F* = 36.685, *p* < 0.001, estimate = 2336.82, SE = 68.01; Additional file 11: Figure S5A) and sex (LMM: DF = 1, *F* = 980.98, *p* < 0.001, estimate = 2634.96, SE = 50.95; Additional file 11: Figure S5A). Female *Ae. albopictus* had an average wing length of 2638.16 ± 0.55 μm and males 2197.18 ± 0.31 μm. Female *Ae. aegypti* had an average wing length of 2914.54 ± 11.26 μm and males 2335.65 ± 7.73 μm (Additional file 11: Figure S5A).

We next analyzed how size changed during the study period by testing the significance of month, collection site, environmental variables and size as predictor variables of *Ae. albopictus* captures. We found that male size significantly changed with the month during the study (LMM: DF = 5, *F* = 2.64, *p* = 0.022, estimate = 2218.82, SE = 23.50; Additional file 11: Figure S5B) but female size did not (LMM: DF = 5, *F* = 1.309, *p* = 0.258, estimate = 2644.75 SE = 37.97). Similarly, we found an effect of collection site (LMM: DF = 18, *F* = 1.855, *p* = 0.01, estimate = 2168.96, SE = 16.184; Additional file 11: Figure S5C) and overhead vegetation cover on male size (LMM: DF = 4, *F* = 3.97, *p* = 0.003, estimate = 2158.22, SE = 13.58; Additional file 11: Figure S5D)—larger males were found in sites with medium vegetation cover. In contrast, female size was not affected by collection site (LMM: DF = 18, *F* = 1.894, *p* = 0.265, estimate = 2619.26, SE = 27.12; Additional file 11: Figure S5C) or vegetation coverage (LMM: DF = 4, *F* = 1.491, *p* = 0.20, estimate = 2649.99, SE = 22.65; Additional file 11: Figure S5D). We also observed a significant association of *Ae. albopictus* size with precipitation, temperature and wind speed for both sexes (Additional file 6: Table S6). Precipitation was directly proportional (Additional file 11: Figure S5E), while temperature and wind speed were both inversely proportional to adult size (Additional file 11: Figure S5F, G).

## Discussion

*Aedes albopictus* is a major vector of arboviruses in several countries [50], a species that has colonized all continents except Antarctica [4] due to its ability to adapt to a wide range of ecological habitats [51]. However, we know little about how climate variables or site characteristics affect its biology and behavior. Further, interactions between *Ae. aegypti* and *Ae. albopictus* have mostly been assessed at the larval level [52–54], with less information regarding how adult population interactions may influence behavior. Mosquito surveillance in tropical urban parks has shown that medically relevant mosquitoes, including *Ae. albopictus*, are often abundant in these spaces [32–34]. We sampled local mosquito populations for 2 years in the Medellín Botanical Garden, an area with high vegetation and human intervened environments representing several micro-ecosystems which makes it an excellent open-field laboratory to test mosquito habitat use and competition.

We found the Medellín Botanical Garden is populated by few mosquito species, with *Ae. albopictus* being the predominant species throughout our study. The size and location of the Botanical Garden likely accounts for this, as small, more centralized parks have lower mosquito richness compared to larger more peripheral parks in other tropical cities [55]. That we collected few *Ae. aegypti* was surprising, as adjacent neighborhoods have high densities of this species (Secretaria de Salud Medellín, unpublished data). Although we collected larvae from natural containers, we found *Ae. aegypti* and *Ae. albopictus* often shared breeding sites, but that *Ae. albopictus* was always found in higher numbers. The Medellín Botanical Garden is well maintained with little peri-domestic containers present in common areas. However, bromeliads, bamboos, palms, and other plants and trees with the ability to act as breeding sites are common around the park. Egg-laying preferences may have played a role in *Ae. albopictus* remaining dominant, as females prefer to oviposit in natural containers [33, 56, 57] and are attracted to sites with existing larvae [58]. This is in contrast to female *Ae. aegypti* preference to oviposit in artificial containers [59]. Our results show that *Ae. aegypti* females will sometimes oviposit in natural breeding sites albeit in low numbers compared to *Ae. albopictus*, similar to studies in other urban parks [33, 57, 60]. Vegetation types may also influence species predominance, as rearing pool detritus can influence larval competition, often favoring *Ae. albopictus* over *Ae. aegypti* [52]. *Aedes albopictus* is frequently associated with peri-urban areas with a high density of vegetation coverage [61], although it also invades urban areas [13, 62] which can increase larval development rates and adult longevity of this species [62], suggesting that urbanization of *Ae. albopictus* populations may increase their vectorial capacity. The Botanical Garden may act as a hotspot for *Ae. albopictus*, as recreational spaces high in vegetation may act as “small green islands” in metropolitan areas [13]. Factors that favor *Ae. albopictus* establishment may be important in disease transmission by this species, particularly in a dense tropical city such as Medellín.

During our study, more adult males were collected despite using human landing catch and sweep nets to capture adults, the opposite to what has been described using this method [63]. This prompted us to examine the spatial distributions of each sex. While *Aedes* population density is correlated with increased vegetation [64], we detected unique spatial distributions of males and females that correlated with overhead vegetation cover. Males were primarily captured in areas with high overhead cover, while females were more evenly distributed. Male and female conspecifics can have similar spatial distributions based on the homogeneous allocation of resources and/or risks that occur at local scales [65]. However, sub-population structures based on local resource competition, such as breeding sites or food sources, may occur [66]. For instance, in polygynous species, males disperse more widely to find receptive females, while females have smaller dispersion ranges [66, 67]. In monogamous species, however, there may be no benefit for differential dispersion patterns of males and females. *Aedes* mosquitoes have polygamous males and monogamous females [46, 68, 69], but we still observed different male–female distribution patterns, suggesting unique factors influence dispersion of *Ae. albopictus* males and female at a local scale. Sex-specific feeding preferences—males feed on nectar while females can feed on nectar and/or blood-feed—may have contributed to the unique spatial distributions we observed. Sites with high vegetation cover often had flowering plants, suggesting that nectar sources may influence male–female distributions at local scale, potentially an important consideration for control programs that release only male mosquitoes [26, 31].

Climatic variables strongly influence population dynamics of *Aedes* mosquitoes [5], and studies conducted in urban parks in tropical cities have found that temperature and rainfall play major roles in the dynamics of urban mosquito populations. Wilke et al. [34] found a predictive association between temperature and accumulated monthly rainfall and mosquito abundance, while Medeiros-Sousa et al. [33] observed a significant relationship between mosquito abundance with warm and rainy periods of their study. Heinisch e Silva et al. [32] also found that seasonal variation in *Aedes* abundance was mediated by environmental temperature, but did not observe a correlation of mosquito abundance with rainfall. Higher temperatures are also associated with *Ae. albopictus* incidence due to optimal conditions for larval rearing with warmer temperatures [9, 50, 70], although *Ae. albopictus* are also resistant to low temperatures [9, 71]. In our study, we found that precipitation was the main environmental factor influencing adult *Ae. albopictus* captures. Rainfall is directly proportional to mosquito density and is associated with *Ae. albopictus* incidence [50]. However, we observed that our adult captures were inversely proportional to temperature. It is possible that the negative correlation might reflect more adult activity at lower temperatures. Flight activity of *Ae. aegypti* is optimal at 21 °C [5], although it is unknown whether *Ae. albopictus* is similar in this regard. The negative correlation may also have been influenced by our collection methods or the time of day when our collections were conducted, as adult *Ae. albopictus* display two peaks of activity during a 24 h period in an outdoor setting—in the morning and in the late afternoon [72]. Our collection time also explains why so few *Culex* spp. adults were captured, as *Culex* are primarily active at night [73, 74].

Body size of adult *Ae. albopictus* was also influenced by vegetation and climatic variables. However, the influence of vegetation on adult size differed by sex. Similar to our adult captures, precipitation was positively correlated with body size and possibly fitness, as larger body size is associated with increased fertility in *Aedes* males and females [46, 75, 76]. However, adult size decreased as temperature increased, possibly due to shorter development times. *Aedes albopictus* females reared at lower temperatures develop into larger adults, with their ovaries displaying higher levels of protein, lipids, and carbohydrates than females reared at higher temperatures, which is suggested to contribute to their increased longevity [77]. Interestingly, overhead vegetation cover influenced male size, but had no effect on female size, although the reason for this difference is unclear. Body size estimations of natural populations, and how environmental factors influence mosquito size, are important parameters to understand interspecific competition and can give baseline information for mosquito control programs that are based on insect release.

## Conclusions

Our study reveals that the Botanical Garden can sustain *Ae. albopictus* populations and further suggests that public spaces high in vegetation can act as hotspots for this species in metropolitan areas [13]. The identification of local factors that favor *Ae. albopictus* establishment, and how these factors influence male–female distributions, will highlight characteristics that influence population dynamics of this species. Rainfall and temperature were significant factors influencing overall *Ae. albopictus* abundance in the Medellín Botanical Garden. However, male and female *Ae. albopictus* had unique local distributions, suggesting that local factors influence how the sexes disperse across the environment; overhead vegetation coverage was the major factor influencing *Ae. albopictus* male distribution, but did not influence females. Whether other mosquito species behave similarly, and whether similar male–female distributions are typical in other urban parks, is an area for further exploration. This study adds to our understanding regarding the roles of environmental variables on *Ae. albopictus* establishment and abundance and identifies local characteristics that may make urban areas susceptible to colonization by this species, which is of particular interest to cities being invaded by *Ae. albopictus*, such as Medellín.

## Supplementary Information

**Additional file 1: Table S1.** Description and overhead vegetation coverage percentage of each collection site. Sites marked with an asterisk (*) denote a larvae collection site.

**Additional file 2: Table S2.** Weather variables registered by SIATA during 2018–2019 in the Medellín Botanical Garden. The data shown is monthly average for each variable.

**Additional file 3: Table S3.** Total and monthly averages of *Ae. aegypti* and *Ae. albopictus* adults and larvae collected at each site during 2018–2019 in the Medellín Botanical Garden. Different letters correspond to significant differences (*p* < 0.05) between the average *Ae. albopictus* collected at each site using a post hoc Tukey-test.

**Additional file 4: Table S4.** Linear mixed model correlations between climate variables as the fixed variables and adult *Ae. albopictus* collections as the response variables in the Medellín Botanical Garden. Table shows the estimates of the y-intercept, regression coefficient of the model, F test statistic used in linear regression and o-value. Statistically significant correlations are shown in **Bold** (*p* < 0.05).

**Additional file 5: Table S5.** Linear mixed model correlations between climate variable and collections of *Ae. aegypti* and *Ae. albopictus* larvae in the Medellín Botanical Garden. Table shows the estimates of the y-intercept, regression coefficient of the model, F test statistic used in linear regression and o-value. Statistically significant correlations are shown in **Bold** (*p* < 0.05).

**Additional file 6: Table S6.** Linear mixed model correlations between climate variables and adult wing size of captured *Ae. albopictus* in the Medellín Botanical Garden. Table shows the estimates of the y-intercept, regression coefficient of the model, F test statistic used in linear regression and o-value. Statistically significant correlations are shown in **Bold** (*p* < 0.05).

**Additional file 7: Figure S1.** Total adult and larvae collections of *Ae. aegypti* and *Ae. albopictus* per site within the Medellin Botanical Garden during the study period. Larvae collections (average ± SE) per site in 2018 (**A**) and 2019 (**B**). Different letters correspond to significant differences with a Tukey-test (*p* < 0.05). Adult Captures (average ± SE) per site in 2018 (**C**) and 2019 (**D**). Tables in **A** – **D** correspond to the summary of the total individuals collected per site in each year.

**Additional file 8: Figure S2.** The average proportion of adult males and females captured at each site in 2018 (**A**) and 2019 (**B**) within the Medellín Botanical Garden.

**Additional file 9: Figure S3.** Seasonal distributions of male and female *Ae. albopictus* in the Medellín Botanical Garden. Male distribution during the (**A**) first (*I*_*a*_ = 1.0622, *P*_*a*_ = 0.3205) and (**B**) second dry season (*I*_*a*_ = 0.7307, *P*_*a*_ = 0.95275), and (**C**) first (*I*_*a*_ = 1.0612, *P*_*a*_ = 0.3185) and (**D**) second rainy season (*I*_*a*_ = 1.0693 Pa = 0.325). Female distribution during the (**E**) first (*I*_*a*_ = 1.1873, *P*_*a*_ = 0.15675) and (**F**) second dry season (*I*_*a*_ = 0.8294, *P*_*a*_ = 0.816), and (**G**) first (*I*_*a*_ = 1.3085, *P*_*a*_ = 0.0615) and (**H**) second rainy season (*I*_*a*_ = 0.8598 Pa = 0.76175). Shaded areas represent local indices of clustering: orange above expectation (*V*_*i*_ > 1), red well above expectation (*V*_*i*_ > 1.5), green below expectation (*V*_*j*_ < -1), and blue well below expectation (*V*_*j*_ < -1.5). Association and disassociation of males and females during the (**I**) first (disassociation *p* = 0.999, association *p* = 0.0004 and (**J**) second dry season (disassociation *p* = 0.8064, association *p* = 0.1936), and (**J**) first (disassociation *p* = 0.9992, association *p* = 0.0008) and (**K**) second rainy season (disassociation *p* = 0.9886, association *p* = 0.0114).

**Additional file 10: Figure S4.** Temporal distribution of male and female *Ae. albopictus* larvae in relation to precipitation during 2018 and 2019.

**Additional file 11: Figure S5.** Wing size analysis of adults collected in the Medellín Botanical Park during the 2018–2019 study period. Wing length of males and females of both *Aedes* species collected (**A)**. Male and female *Ae. albopictus* wing lengths per month of each study year (**B),** per site of collection site (**C**), and at sites with the corresponding vegetation coverage (**D**). Linear model fit between precipitation (**E**), temperature (**F**), and wind speed (**G**) and male and female wing size during the 2 years of study.

## Data Availability

The datasets generated during and/or analyzed during the current study are available from the corresponding authors upon reasonable request.

## Abbreviations

AIC: Akaike information criterion
SE: Standard error
LMM: Linear mixed model
GLMM: Generalized linear mixed model
SADIE: Spatial analysis by distance indices
SIATA: Sistema de Alerta Temprana de Medellín y el Valle de Aburrá (i.e., Early Alert System of Medellín and the Aburrá Valley).
IDW: Inverse distance weighting
