# Supplementary figures and images for: Spatial and temporal population dynamics of male and female Aedes albopictus at a local scale in Medellín, Colombia
Source: Parasit Vectors. 2021 Jun 8;14:312. doi: 10.1186/s13071-021-04806-2 (PMC8188797; doi:10.1186/s13071-021-04806-2)

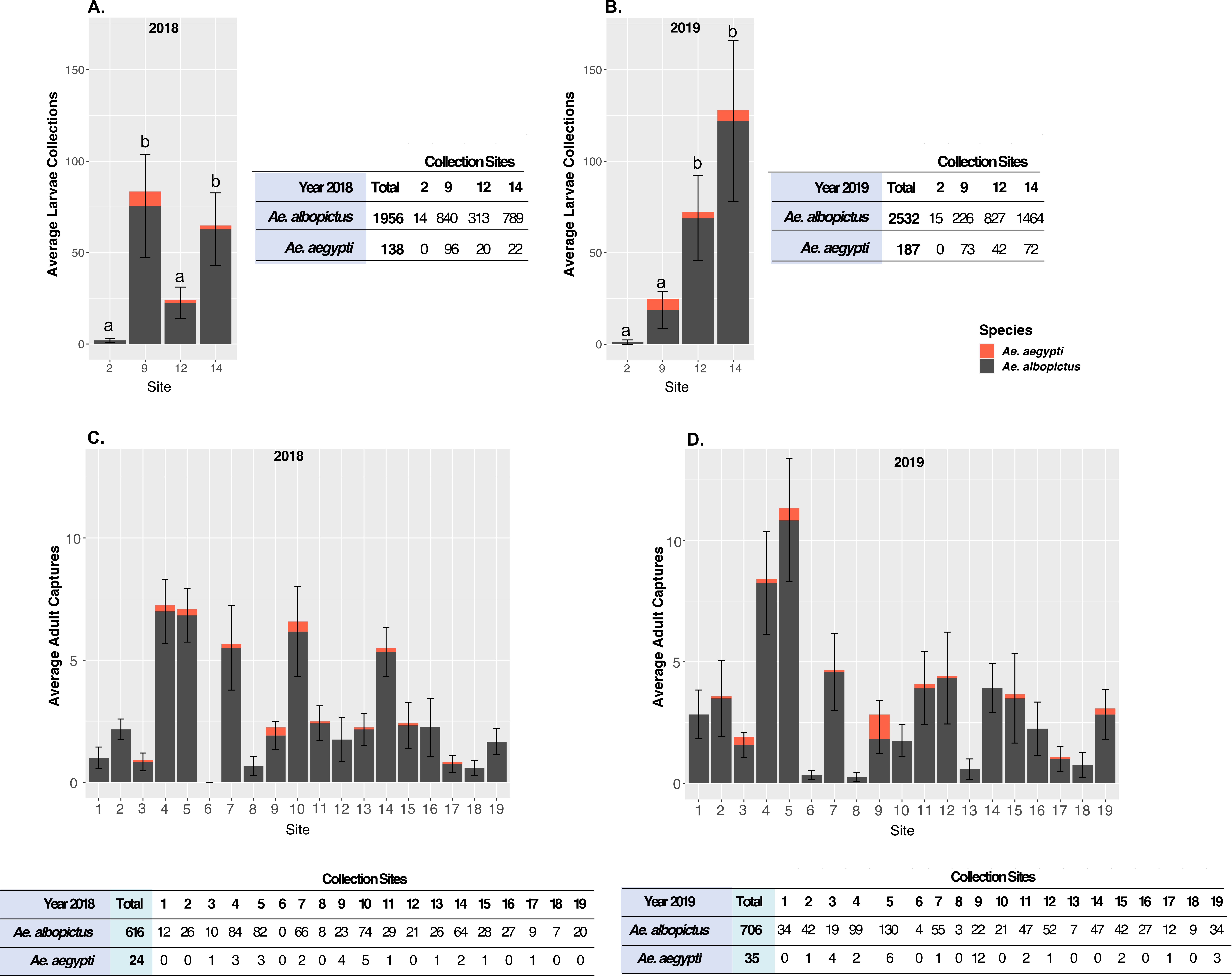

Supplement: Supplementary file 7 — Additional file 7: Figure S1. Total adult and larvae collections of Ae. aegypti and Ae. albopictus per site within the Medellin Botanical Garden during the study period. Larvae collections (average ± SE) per site in 2018 (A) and 2019 (B). Different letters correspond to significant differences with a Tukey-test (p < 0.05). Adult Captures (average ± SE) per site in 2018 (C) and 2019 (D). Tables in A – D correspond to the summary of the total individuals collected per site in each year. [file 13071_2021_4806_MOESM7_ESM.jpg]

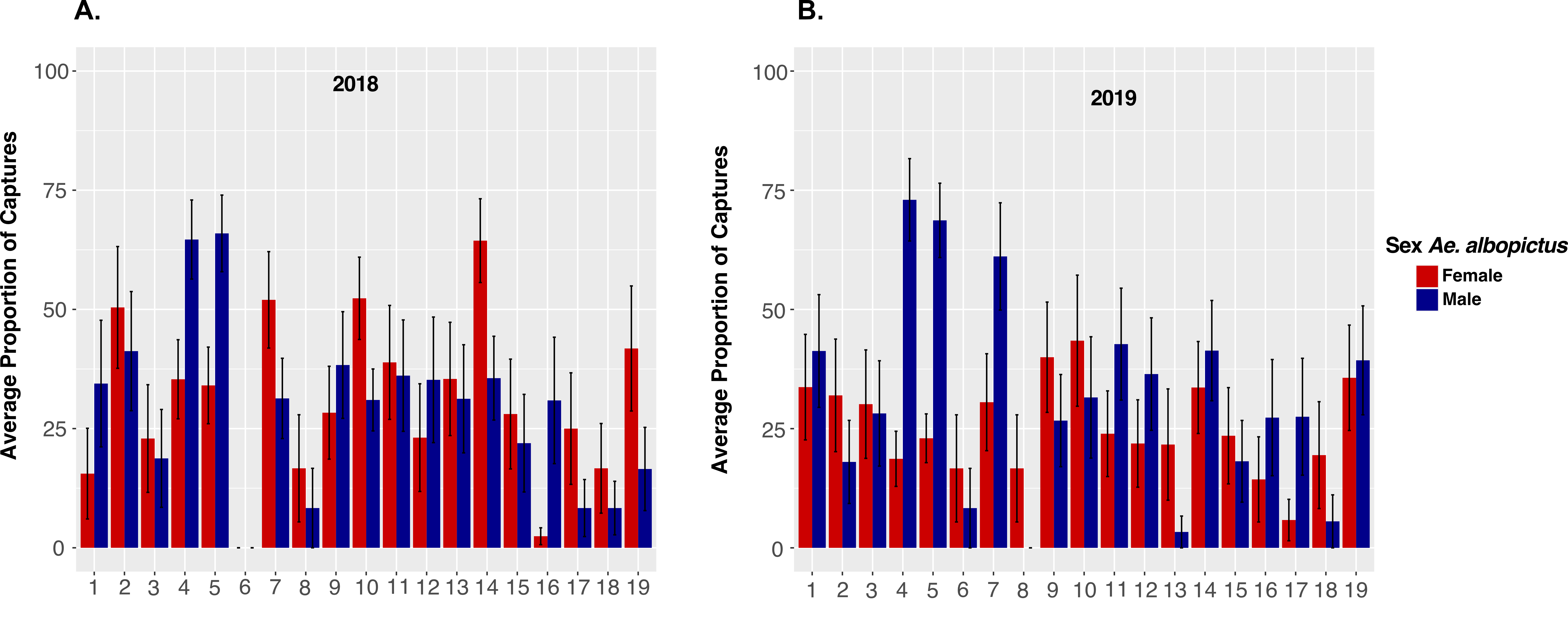

Supplement: Supplementary file 8 — Additional file 8: Figure S2. The average proportion of adult males and females captured at each site in 2018 (A) and 2019 (B) within the Medellín Botanical Garden. [file 13071_2021_4806_MOESM8_ESM.jpg]

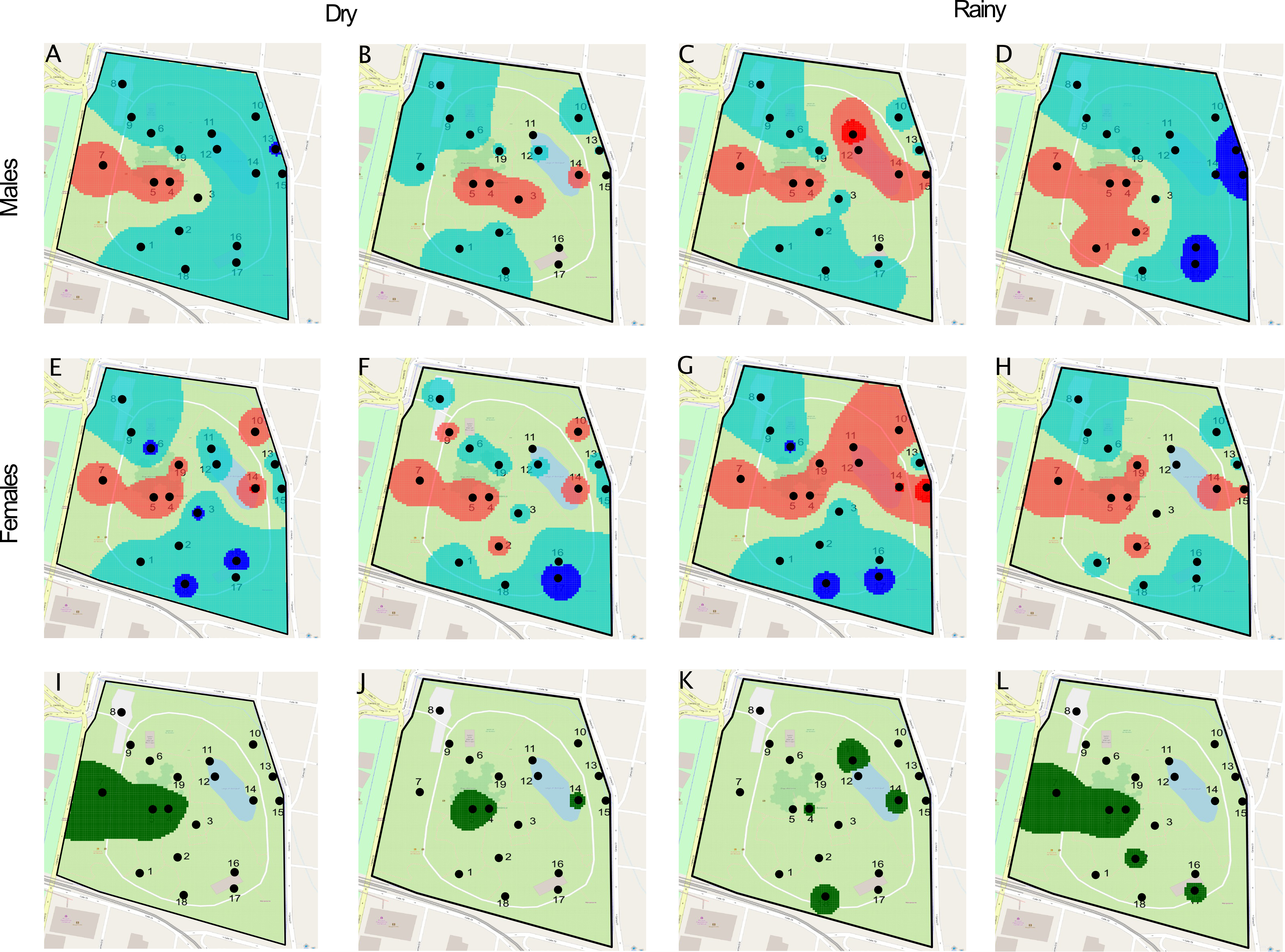

Supplement: Supplementary file 9 — Additional file 9: Figure S3. Seasonal distributions of male and female Ae. albopictus in the Medellín Botanical Garden. Male distribution during the (A) first (Ia = 1.0622, Pa = 0.3205) and (B) second dry season (Ia = 0.7307, Pa = 0.95275), and (C) first (Ia = 1.0612, Pa = 0.3185) and (D) second rainy season (Ia = 1.0693 Pa = 0.325). Female distribution during the (E) first (Ia = 1.1873, Pa = 0.15675) and (F) second dry season (Ia = 0.8294, Pa = 0.816), and (G) first (Ia = 1.3085, Pa = 0.0615) and (H) second rainy season (Ia = 0.8598 Pa = 0.76175). Shaded areas represent local indices of clustering: orange above expectation (Vi > 1), red well above expectation (Vi > 1.5), green below expectation (Vj < -1), and blue well below expectation (Vj < -1.5). Association and disassociation of males and females during the (I) first (disassociation p = 0.999, association p = 0.0004 and (J) second dry season (disassociation p = 0.8064, association p = 0.1936), and (J) first (disassociation p = 0.9992, association p = 0.0008) and (K) second rainy season (disassociation p = 0.9886, association p = 0.0114). [file 13071_2021_4806_MOESM9_ESM.jpg]

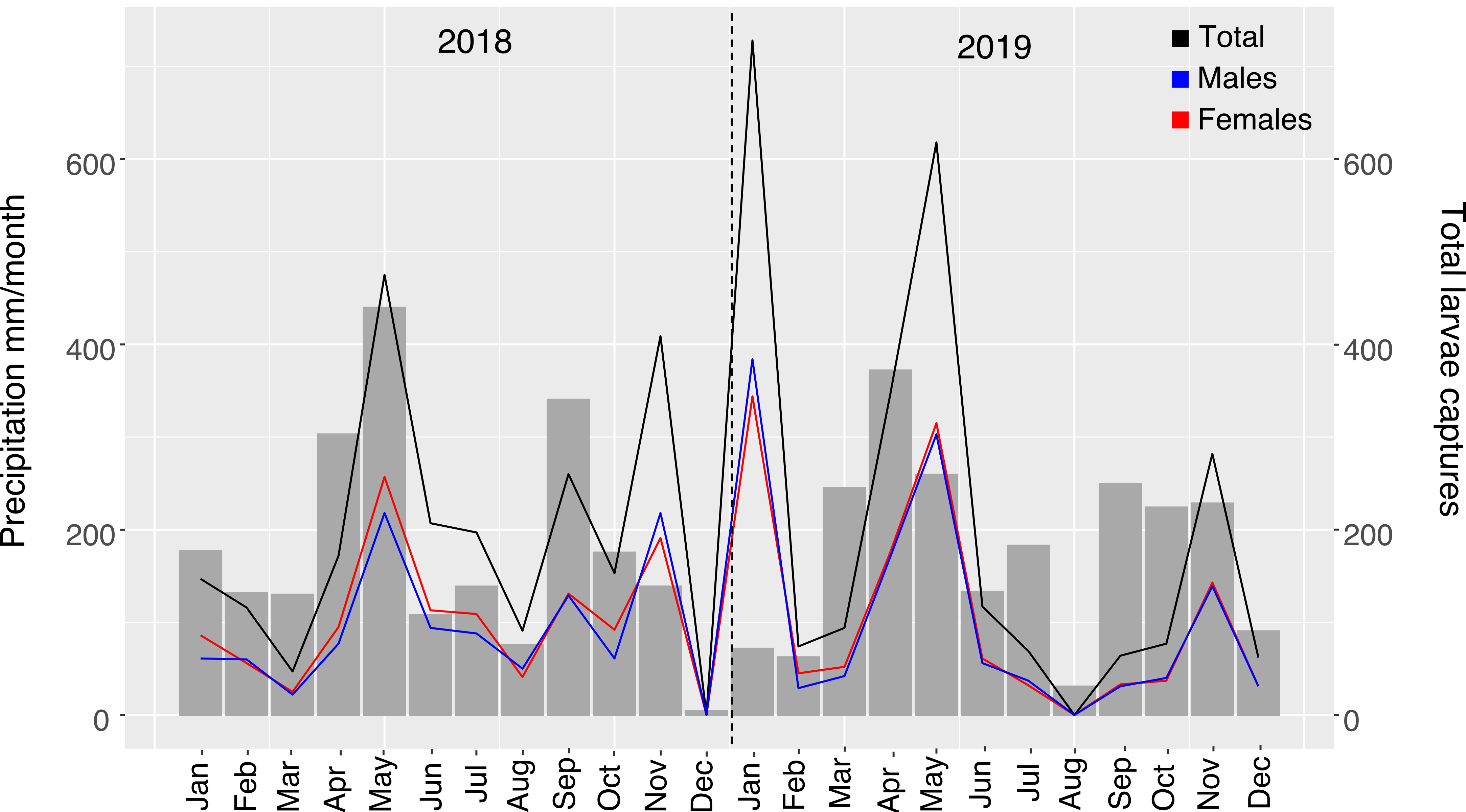

Supplement: Supplementary file 10 — Additional file 10: Figure S4. Temporal distribution of male and female Ae. albopictus larvae in relation to precipitation during 2018 and 2019. [file 13071_2021_4806_MOESM10_ESM.jpg]

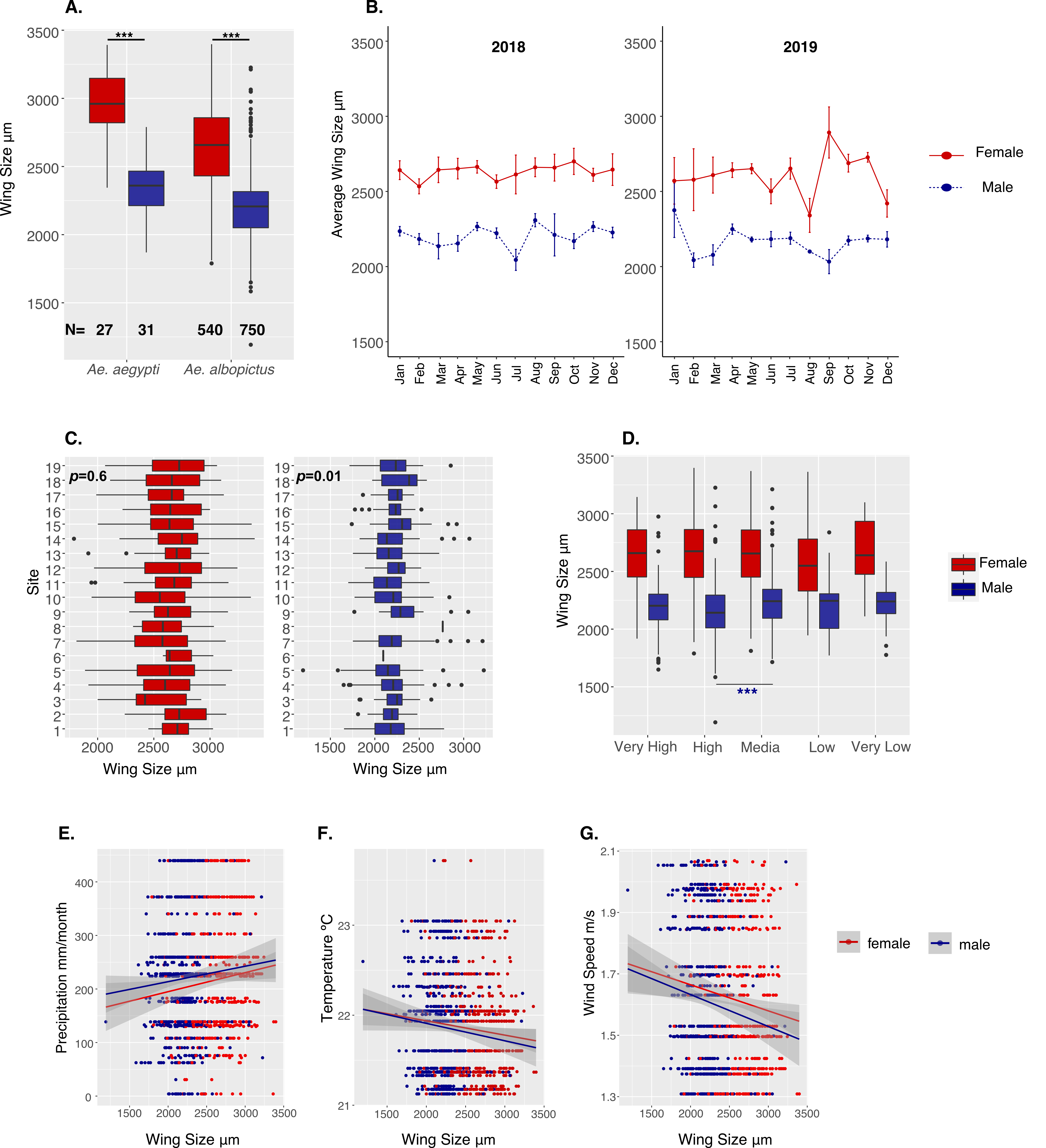

Supplement: Supplementary file 11 — Additional file 11: Figure S5. Wing size analysis of adults collected in the Medellín Botanical Park during the 2018–2019 study period. Wing length of males and females of both Aedes species collected (A). Male and female Ae. albopictus wing lengths per month of each study year (B), per site of collection site (C), and at sites with the corresponding vegetation coverage (D). Linear model fit between precipitation (E), temperature (F), and wind speed (G) and male and female wing size during the 2 years of study. [file 13071_2021_4806_MOESM11_ESM.jpg]
